# Nimotuzumab combined with chemoradiotherapy for the treatment of cervical cancer: A meta-analysis of randomized controlled trials

**DOI:** 10.3389/fonc.2022.994726

**Published:** 2022-10-03

**Authors:** Yan Yuan, Jiuzhou Chen, Miao Fang, Yaru Guo, Xueqing Sun, Dehong Yu, Yilong Guo, Yong Xin

**Affiliations:** ^1^ Department of Radiation, the Affiliated Hospital of Xuzhou Medical University, Xuzhou, China; ^2^ Department of Cancer Institute, Xuzhou Medical University, Xuzhou, China; ^3^ Department of Radiation, the Affiliated Pizhou Hospital of Xuzhou Medical University, Xuzhou, China

**Keywords:** cervical cancer, nimotuzumab, chemoradiotherapy, chemotherapy, radiotherapy, meta-analysis

## Abstract

**Objectives:**

To assess the clinical efficacy and toxicity of nimotuzumab in combination with chemoradiotherapy or chemoradiotherapy alone in the treatment of cervical cancer.

**Methods:**

The PubMed, Embase, Cochrane Library, Web of Science, China National Knowledge Infrastructure, China Biomedical Medicine, Wanfang, and VIP databases were systematically searched for relevant literature. Ultimately, six randomised controlled trials (n=393) were included in our meta-analysis.

**Results:**

A total of 393 patients were included, of which 197 were in the nimotuzumab combined with chemoradiotherapy group and 196 were in the chemoradiotherapy group. The results of our meta-analysis showed that the complete remission rate (risk ratio [RR] = 1.34, 95% confidence interval [CI]: 1.08-1.65, P = 0.007), objective response rate (RR = 1.30, 95% CI: 1.16-1.44, P < 0.05), and three-year survival rate (RR = 1.27, 95% CI: 1.06-1.51, P = 0.008) in the nimotuzumab combined with chemoradiotherapy group were significantly improved compared with the chemoradiotherapy group. This difference was not statistically significant when comparing the incidence of adverse reactions (such as leukocytopenia, gastrointestinal reaction, radiocystitis, and radioproctitis) between the two groups.

**Conclusions:**

Nimotuzumab in combination with chemoradiotherapy has some advantages over chemoradiotherapy alone in the treatment of cervical cancer and does not increase toxicity. Therefore, nimotuzumab has the potential to be an effective treatment for cervical cancer; however, further evidence from large-scale randomised controlled trials is needed.

## 1 Introduction

Cervical cancer (CC) is a common malignancy, ranking fourth in both cancer morbidity and mortality in women, after breast, colorectal, and lung cancers. In 2020, there were approximately 600,000 new cases of CC and 350,000 related deaths worldwide (1). CC is mainly associated with a persistent human papillomavirus (HPV) infection, which causes 99.7% cervical cancers (2, 3). In high-income countries, with the promotion of the CC vaccine and the common use of regular screening of tumours, the incidence and mortality rates of CC have dropped significantly. However, approximately 83% of new cases and 88% of deaths from CC occur in low- and middle-income countries, in which HPV vaccination programs and organised screening are absent (4, 5).

The clinical stage and pathological type of CC determine the treatment methods required by patients, including surgery, radiotherapy, and chemotherapy (6). For patients of CC with FIGO-stages IA-IB2, surgery is the main modality for treatment. Carboplatin/paclitaxel and cisplatin/paclitaxel with or without bevacizumab are the mainstays of treatment for metastatic disease. The standard treatment for patients with locally advanced cervical cancer (LACC) is concurrent chemoradiotherapy (CCRT), and includes external radiation radiotherapy, platinum-based chemotherapy, and brachytherapy (7). However, at least one-third of patients with LACC experience treatment failure, even after receiving platinum-based CCRT (8).

Overexpression of the epidermal growth factor receptor (EGFR) is significantly associated with poor prognosis in patients with malignancies (9). Nimotuzumab is a humanised IgG1 monoclonal antibody that acts on the extracellular structural domain of EGFR and exerts its antitumour effects mainly by inhibiting tumour cell proliferation, anti-angiogenesis, and promoting apoptosis (10). Owing to its low affinity for target cells, nimotuzumab binds preferentially to tumour cells that overexpress EGFR, thereby reducing the effect on normal cells with low levels of EGFR; thus, nimotuzumab has few serious adverse effects, specifically rashes (11). Nimotuzumab has been approved for the treatment of head and neck malignancies and gliomas in several countries, and some clinical trials on CC are underway (12–14). A single-arm study assessed the efficacy and safety of nimotuzumab in combination with single-agent chemotherapy as a second-, third-, or further-line therapy in 17 patients with refractory or progressive CC, and observed no complete remission (CR) or partial remission (PR), with a stable disease (SD) rate of 35%, and median progression-free survival (PFS) and overall survival (OS) of 163 and 299 days, respectively (15). It has been demonstrated that a combination of anti-EGFR monoclonal antibody and radiotherapy enhances the antitumour effect by increasing the cytotoxic effect of radiation and enhancing the inhibition of EGFR signalling (16). Another single-arm study by Qing et al. also showed that neoadjuvant chemoradiotherapy (CRT) plus nimotuzumab combined with surgery was safe for patients with LACC and had good long-term outcomes (17).

Nimotuzumab has been shown to be effective in the treatment of CC, and fewer adverse effects have been reported; however, it remains unclear whether the combination of nimotuzumab with chemoradiotherapy improves the efficacy of CC treatment compared to chemoradiotherapy alone, and whether there is an increase in adverse effects. Therefore, we conducted this meta-analysis to evaluate the efficacy and safety of nimotuzumab combined with CRT compared to CRT alone for CC and to provide more evidence for the treatment of CC.

## 2 Materials and methods

This systematic review and meta-analysis was registered on Inplasy.com (INPLASY protocol 202240098. DOI:10.37766/inplasy2022.4.0098). It was strictly implemented based on the guidelines of the Preferred Reporting Items for Systematic Reviews and Meta-Analysis (PRISMA) (18). As the analysis was based on previously published literature, patient consent and ethical approval were not required.

### 2.1 Inclusion criteria

(i) Definitive diagnosis of CC, including squamous carcinoma, adenocarcinoma, and adenosquamous carcinoma, using puncture cytology or histopathology.
(ii) Comparison of the efficacy and safety of nimotuzumab in combination with CRT versus CRT alone.
(iii) At least one of the following were reported: complete remission rate (CRR), partial remission rate (PRR), objective response rate (ORR), survival rate, or adverse event. The CRR, PRR, ORR, and survival rates were used as the primary efficacy outcomes. The secondary indicators were the incidence of adverse reactions, mainly leukocytopenia, gastrointestinal reactions, radiocystitis, and radioproctitis. The efficacy of the two treatment groups was assessed using the Response Evaluation Criteria in Solid Tumours (RECIST): CR, disappearance of all target lesions; PR, at least 30% reduction in the total diameter of the target lesion; Progressive disease (PD), a minimum 20% increase in the total diameter of the target lesions using the smallest sum as the study reference; SD, insufficient shrinkage to meet PR and insufficient increase to meet PD; and ORR, the proportion of patients whose tumours shrunk to a certain extent and stayed for a certain length of time, including CR and PR cases.
(iv) The study design was a randomised controlled trial (RCT).

### 2.2 Exclusion criteria

(i) Patients with severe heart, lung, liver, or kidney diseases (including coronary atherosclerotic heart disease, pneumonia, liver cirrhosis, and chronic kidney disease).
(ii) The article was a review, meta-analysis, conference abstract, or case report.
(iii) The study was a non-randomised controlled trial (nRCT) or observational study.
(iv) Studies for which data were not available, studies for which no valid data were available, or ongoing clinical trials for which the results were not published.
(v) Unreasonable test design.

### 2.3 Search strategy and study selection

The Cochrane Library, PubMed, Embase, Web of Science, Chinese National Knowledge Infrastructure (CNKI), Chinese Biological Medicine (CBM), Wanfang, and VIP databases were systematically searched for relevant literature from the time of creation of each database to March 2022. Two investigators (YY and JC) independently performed searches using the following terms and their synonyms: uterine cervical neoplasms, nimotuzumab, chemoradiotherapy, chemotherapy, and radiotherapy. Trials were also searched for on the International Clinical Trials Registry Platform (ICTRP) and Chinese Clinical Registry. The searched literature was rigorously screened against the inclusion and exclusion criteria, and those that met these criteria were selected. If there was a dispute, a group discussion with a third determined whether inclusion was necessary. We also manually searched the bibliographies of relevant reviews and trials that met the criteria to identify other relevant articles.

### 2.4 Data extraction and quality assessment

Two investigators (YY and JC) independently extracted relevant data from all eligible studies, including the first author, year of publication, number of patients, Federation International of Gynaecology and Obstetrics (FIGO) stage, patient age, nimotuzumab dose, radiotherapy modality, radiotherapy target area, radiotherapy dose, and chemotherapy regimen. In addition, primary and secondary outcome indicators were extracted, including the CRR, PRR, ORR, survival rate, and adverse events. Any disagreement was resolved through discussion and negotiation between parties. Two investigators (MF and XS) independently evaluated the quality of the final included literature according to the Cochrane Collaboration’s tool for randomized controlled trials, including random sequence generation (selection bias), allocation concealment (selection bias), blinding of participants and personnel (performance bias), incomplete outcome data (attrition bias), selective reporting (reporting bias) and other biases, and assessing each risk of bias as high, low, or unclear risk (19). We used Review Manager 5.3 to graph and evaluate the results. Any disagreements arising from the process of data extraction and quality assessment were discussed and resolved by mutual agreement between the researchers.

### 2.5 Statistical analysis

All meta-analyses were performed using Cochrane RevMan (version 5.3) and Stata (version 16). All results were reported *via* pooled risk ratios (RRs) and 95% confidence intervals (CIs), specifically in the form of forest plots. P-values were all bilateral, and statistical significance was set at P < 0.05. We used the Cochrane Q test and I^2^ statistic to assess the heterogeneity of all included studies. If the heterogeneity was not significant (P > 0.1, I^2^ < 50.0%), a fixed-effects model was used; otherwise, a random-effects model was used. We evaluated potential publication bias using funnel plots, Egger’s test, and Begg’s test, with P > 0.05 indicating no potential publication bias.

## 3 Results

### 3.1 Characteristics of studies

**Figure 1:**
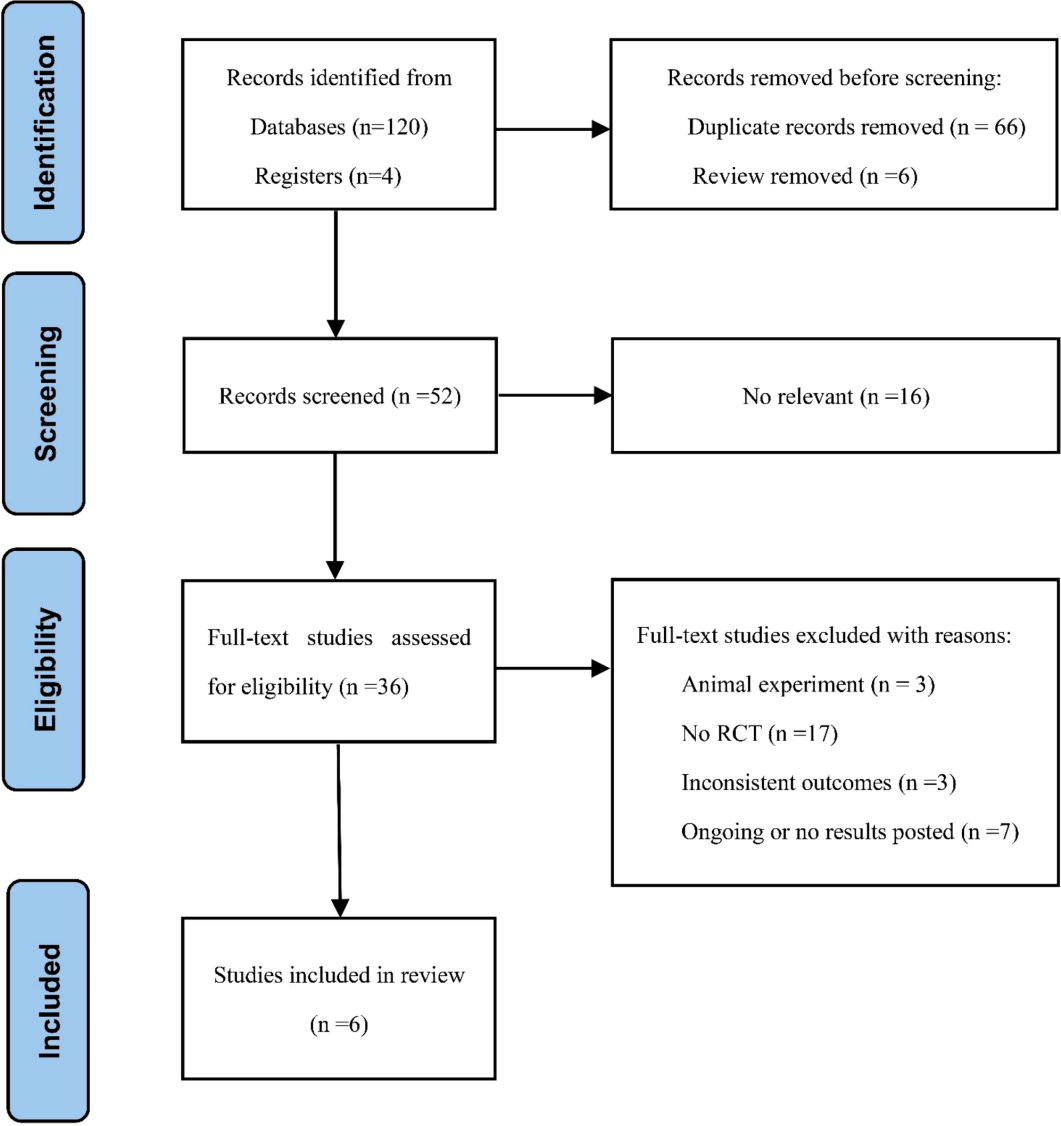
Flow chart of the literature selection process.

**Figure 1** shows a detailed flow chart of the literature screening process. We preliminarily identified 120 relevant articles from the databases and found four trials from the registries, of which 66 articles were repeated. We then evaluated and screened the titles, abstracts, or full texts in strict accordance with the inclusion and exclusion criteria, and finally included six qualified RCTs for meta-analysis (20–25). Finally, the complete articles included were obtained from the database. **Table 1** summarises the characteristics of the articles in detail. A total of 393 patients were finally included: 197 (50.1%) in the experimental group (nimotuzumab plus CRT group) and 196 (49.4%) in the control group (CRT group). These studies were published from 2015 to 2021, and the FIGO stage of the patients was Ib3-IV. In the included studies, the treatment of the control group was CCRT, of which five (20–24) used intensity-modulated radiation therapy (IMRT), one (25) did not mention the specific radiotherapy method, and four (20–22, 24) used intracavitary radiotherapy. Platinum-based chemotherapy was used in all studies, of which four (20, 22, 23, 25) were treated with cisplatin, one (21) with nedaplatin, and one (24) with the TP regimen. The experimental group was treated with nimotuzumab according to the treatment scheme of the control group.

**Table 1:** Characteristics of studies included.

Study	Study design	Sample size (Exp/Con)	Stage	Age/years		Nimotuzumab dose	Radiotherapy		chemotherapy
				Exp	Con		Radiotherapy types	Target area and radiation dose	
Cao Y 2019 (20)	RCT	46/46	IIb-IVa	55.43 ± 10.10	57.08 ± 9.91	200mg/week/6 weeks	IMRT	Pelvic radiotherapy: 50~55 Gy Intracavitary radiotherapy: 5Gy/week	Cisplatin: 40mg/m^2^/week/6 weeks
Chen YF 2015 (21)	RCT	36/36	IIb-IVa	54.2 ± 11.8	55.2 ± 11.5	200mg/week	IMRT	Pelvic radiotherapy: average dose:54.5 Gy Intracavitary radiotherapy: 5Gy/week/4-5 weeks	Nedaplatin: 40 mg/m^2^/week
Sun MH 2020 (22)	RCT	37/37	Ib3-IVa	18~75	18~75	400mg/week/6 weeks	IMRT	Pelvic radiotherapy: 50.4 Gy Intracavitary radiotherapy: 30 Gy	Cisplatin: 40mg/m^2^/week/6 weeks
Tian LC 2021 (23)	RCT	27/27	IIb-IV	54.80 ± 2.13	54.19 ± 2.07	200mg/week	IMRT	Pelvic radiotherapy: 45~50.4 Gy	Cisplatin: 40 mg/m^2^/week
Yan HW 2021 (24)	RCT	21/20	III-IVa	49.31 ± 9.02	47.63 ± 8.79	100mg/week	IMRT	Pelvic radiotherapy: 50~55 Gy Metastatic lymph node: 59.92Gy Intracavitary radiotherapy	Paclitaxel: 135 mg/m^2^/week Cisplatin: 75 mg/m^2^/week
Zheng WT 2018 (25)	RCT	30/30	IIb-IV	49.52 ± 0.79	48.42 ± 0.58	200mg/week/6 weeks	NA	Pelvic radiotherapy: 50~55 Gy Metastatic lymph node:60Gy	Cisplatin: 40mg/m^2^/week/6 weeks

RCT, randomized controlled trial; IMRT, intensity modulated radiation therapy; NA, not available.

### 3.2 Quality assessment

**Figure 2:**
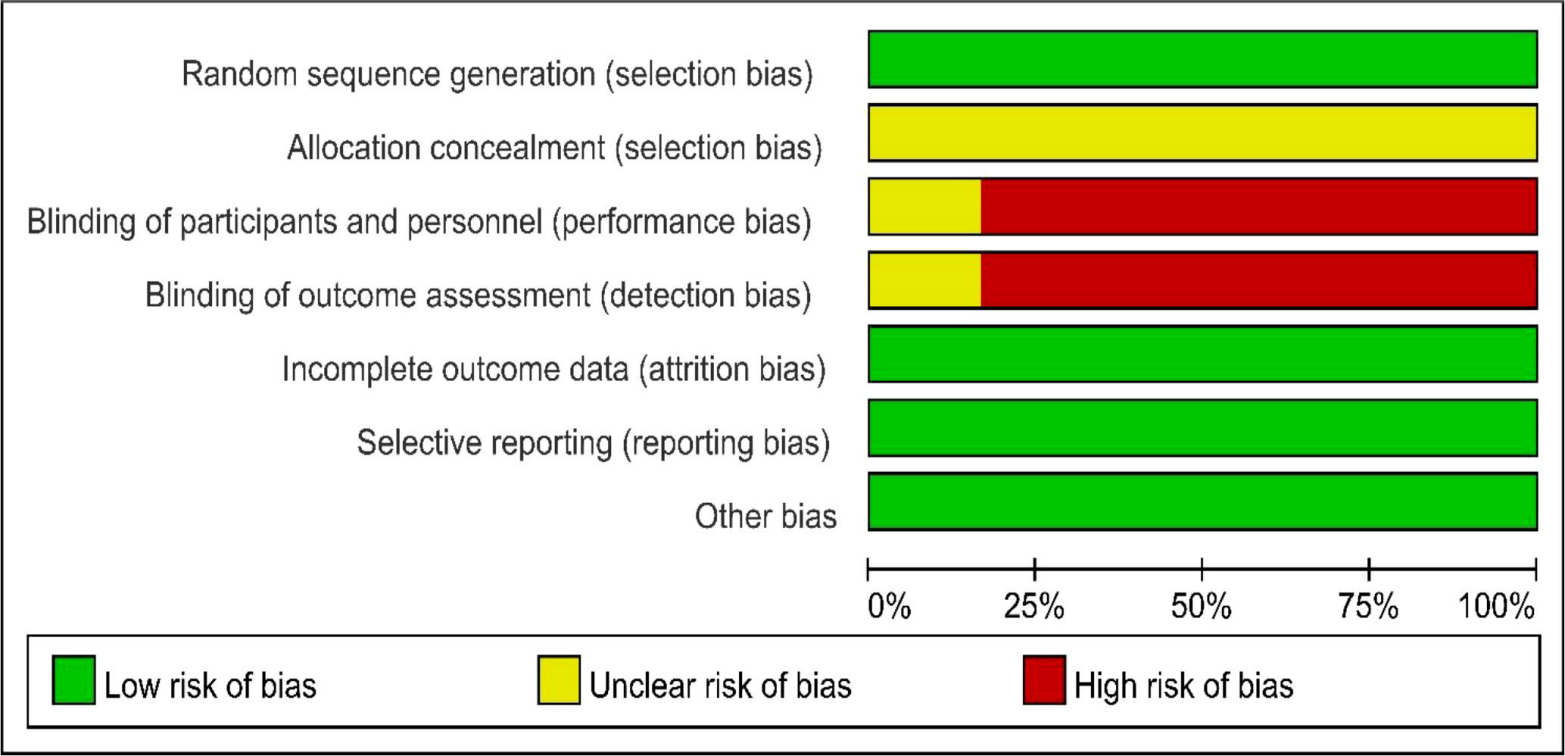
Risk of bias assessment.

**Figure 3:**
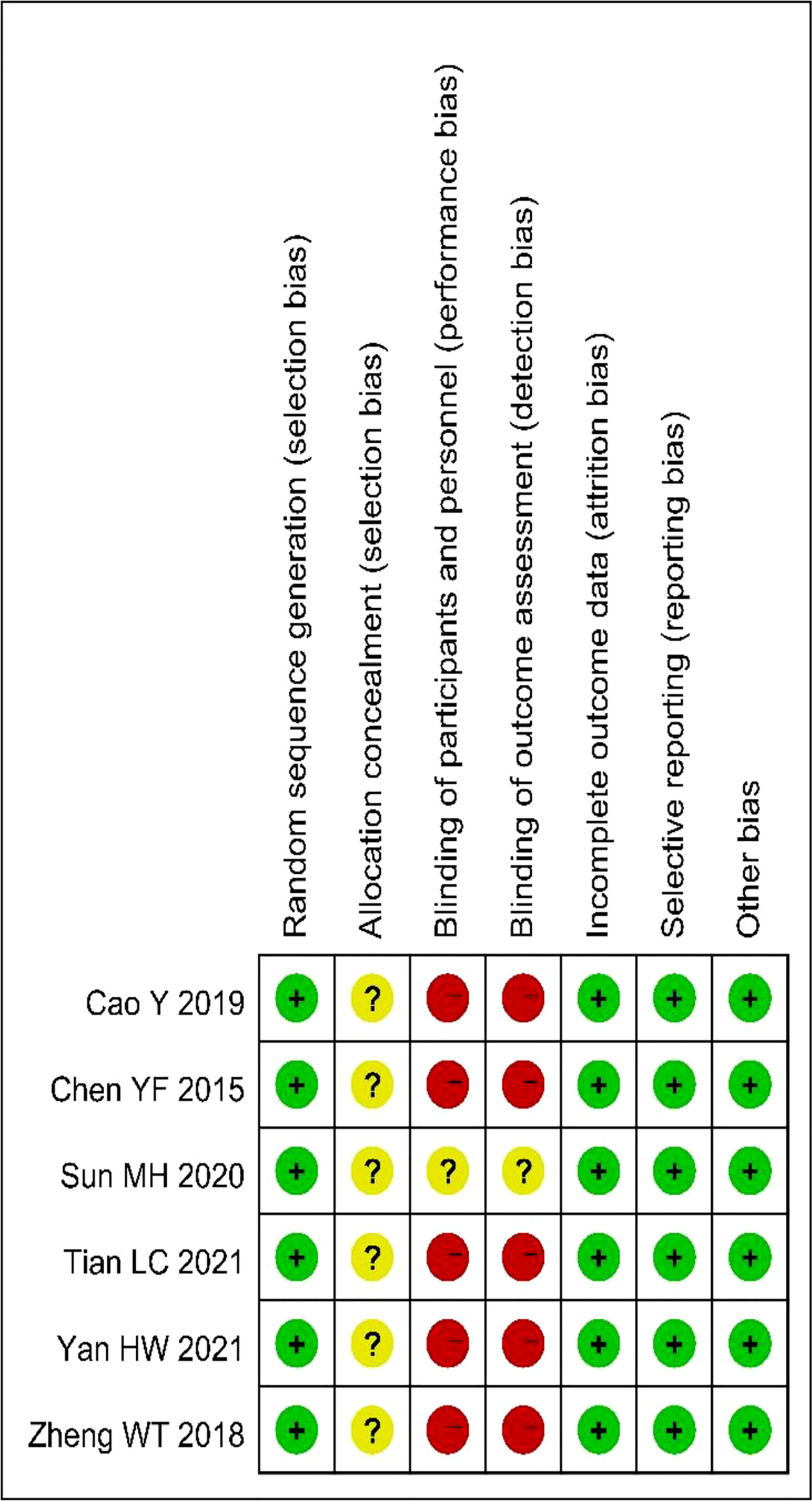
Risk of bias summary.

As shown in **Figures 2** and **3**, we evaluated the quality of all final included studies using the Cochrane Collaboration’s tool. All the studies finally included were RCTs. Five (20, 22–25) were randomly assigned using the random numbers method, while the other one (21) did not describe any specific random method. None of the included studies provided sufficient information to evaluate whether the allocation concealment was sufficient. Five studies (20, 21, 23–25) obtained informed consent from all included patients and were considered to be non-blinded. The remaining study (22) did not mention relevant information on blinding. The data and reports of the results included in the study were complete, without selective reporting or other biases. Overall, the risk of bias was low in all the studies included in this meta-analysis.

### 3.3 Efficiency

#### 3.3.1 CRR

**Figure 4:**
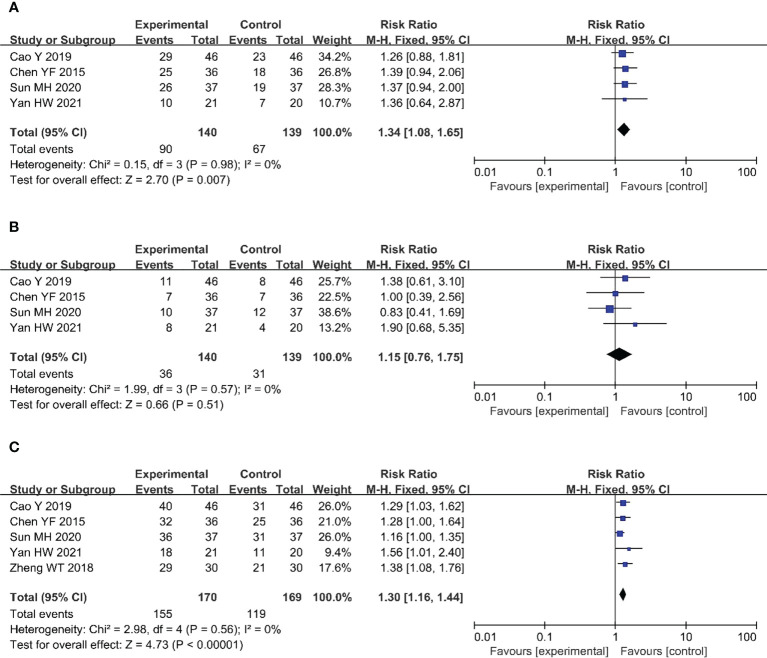
Forest plot for CRR **(A)**, PRR **(B)**, ORR **(C)** of nimotuzumab combined with CRT group and CRT alone group. CRR, complete remission rate; PRR, partial remission rate; ORR, objective response rate; CRT, chemoradiotherapy.

Four (20–22, 24) of the included studies (279 patients) reported CRRs in patients with CC. There was no heterogeneity among the studies (P = 0.98, I^2^ = 0%); therefore, we used a fixed-effects model for the analysis. The results showed that compared with the CRT group, the CRR of the nimotuzumab combined with CRT group was significantly higher (RR = 1.34, 95% CI: 1.08-1.65, P = 0.007 < 0.05) (**Figure 4A**).

#### 3.3.2 PRR

Four (20–22, 24) studies (279 patients) reported PRRs in patients with CC. There was no heterogeneity among the studies (P = 0.57, I^2^ = 0%); therefore, we used a fixed-effects model for the analysis. This result was not statistically significant (RR = 1.15, 95% CI: 0.76-1.75, P = 0.51 > 0.05) (**Figure 4B**).

#### 3.3.3 ORR

Five (20–22, 24, 25) of the included studies (339 patients) reported ORRs in patients with CC. There was no heterogeneity among the studies (P = 0.56, I^2^ = 0%); therefore, we used a fixed-effects model for the analysis. The results showed that, compared with the CRT group, the ORR of the nimotuzumab combined with CRT group was significantly higher (RR = 1.30, 95% CI: 1.16-1.44, P < 0.05) (**Figure 4C**).

### 3.4 Three-year survival rate

**Figure 5:**
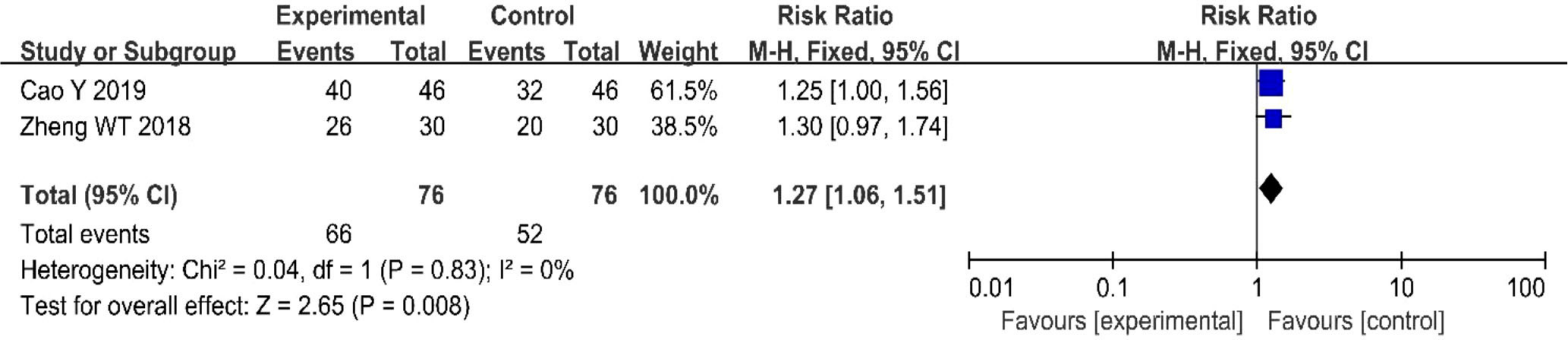
Forest plot for 3-year survival rates.

Two (20, 25) of the included studies (152 patients) reported three-year survival rates in patients with CC. There was no heterogeneity among the studies (P = 0.83, I^2^ = 0%); therefore, we used a fixed-effects model for the analysis. The results showed that, compared with the CRT group, the three-year survival rate of the nimotuzumab combined with CRT group was significantly higher (RR = 1.27, 95% CI: 1.06-1.51, P = 0.008 < 0.05) (**Figure 5**).

### 3.5 Adverse effects

**Figure 6:**
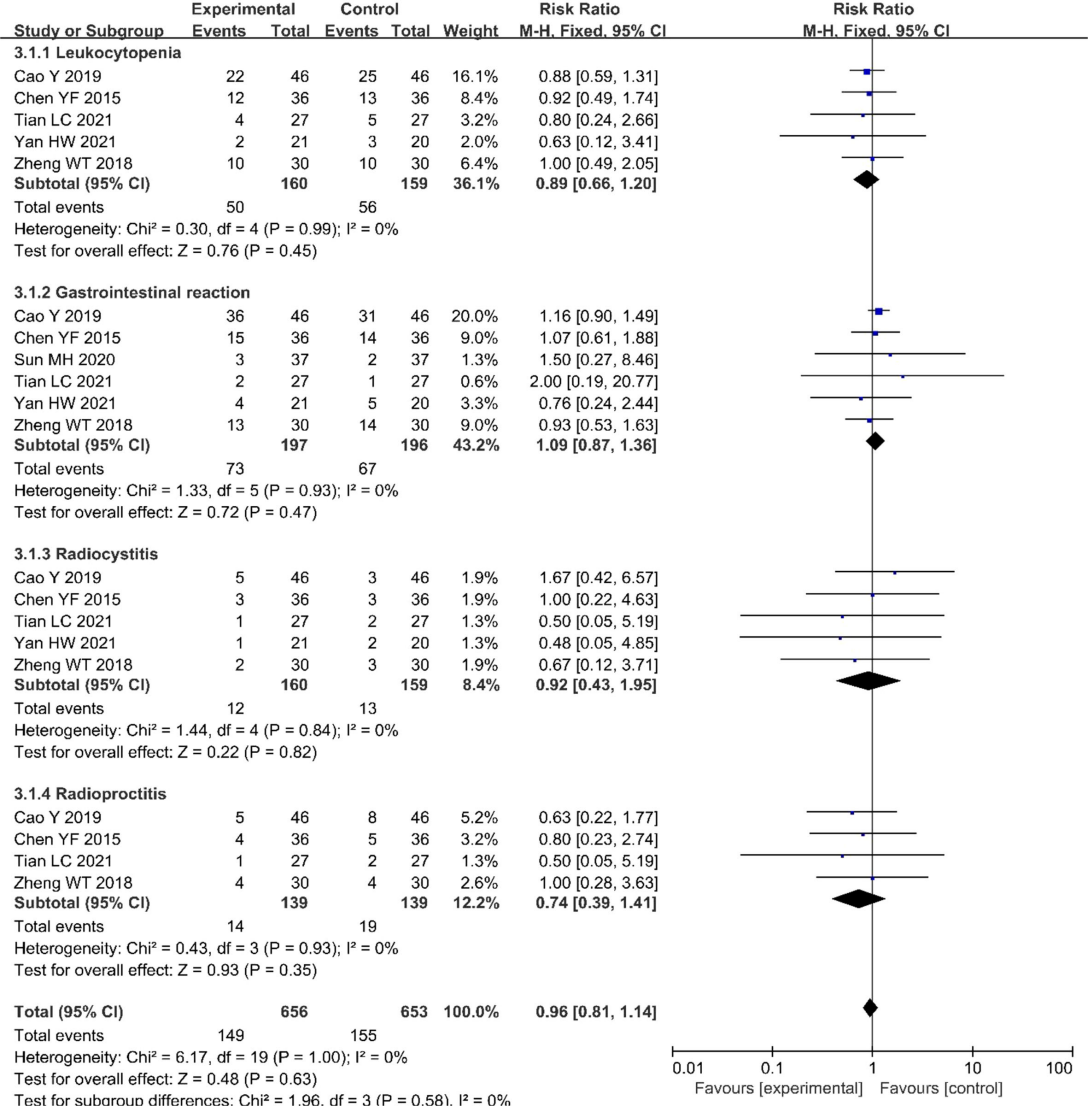
Forest plot for adverse reactions of nimotuzumab combined with CRT group and CRT alone group. CRT, chemoradiotherapy.

Five (20, 21, 23–25) of the included studies (319 patients) reported leukocytopenia, six (20–25) (393 patients) reported three gastrointestinal reactions, five (20, 21, 23–25) (319 patients) reported radiocystitis, and four (20, 21, 23, 25) (278 patients) reported radioproctitis. All adverse reaction incidences showed no significant differences between the nimotuzumab combined with CRT group and the CRT group (**Figure 6**).

### 3.6 Evaluation of the sensitivity and publication bias

**Figure 7:**
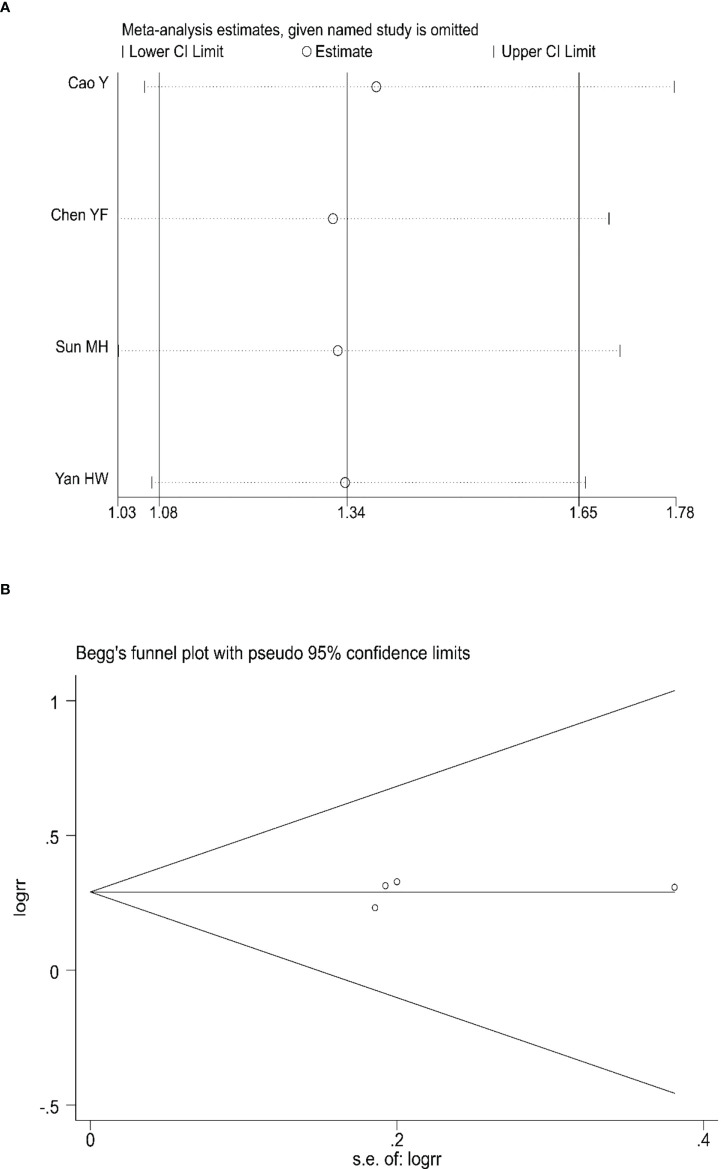
Sensitivity analysis **(A)** and Begg’s funnel plot **(B)** for the analysis of CRR. (Begg’s test: p = 0.734). CRR, complete remission rate.

**Figure 8:**
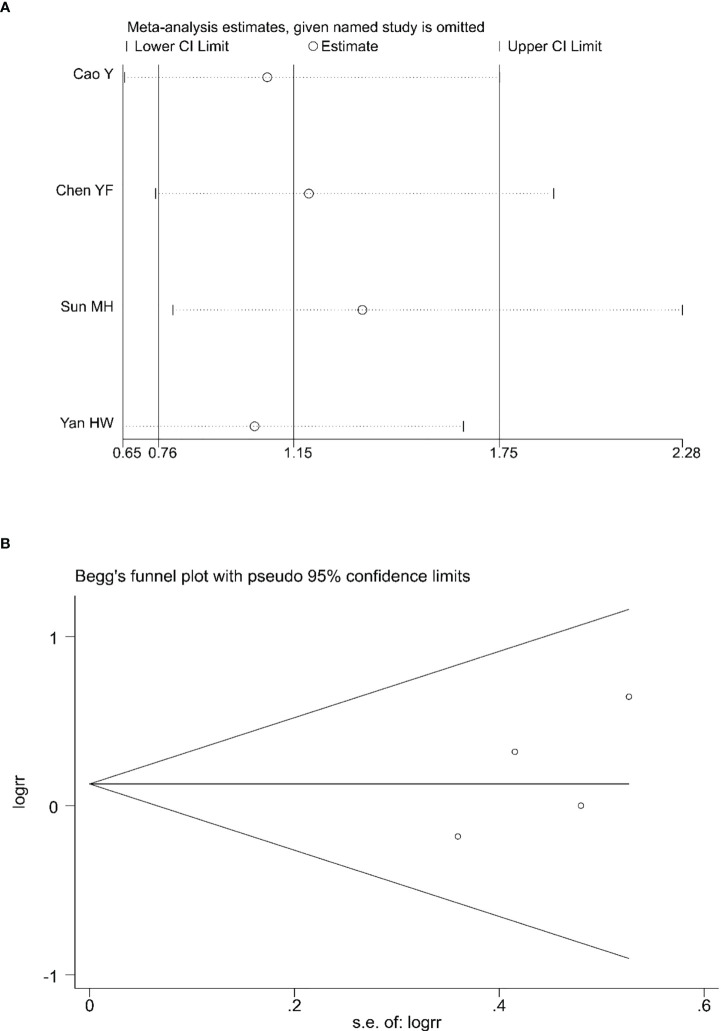
Sensitivity analysis **(A)** and Begg’s funnel plot **(B)** for the analysis of PRR. (Begg’s test: p = 0.308). PRR, partial remission rate.

**Figure 9:**
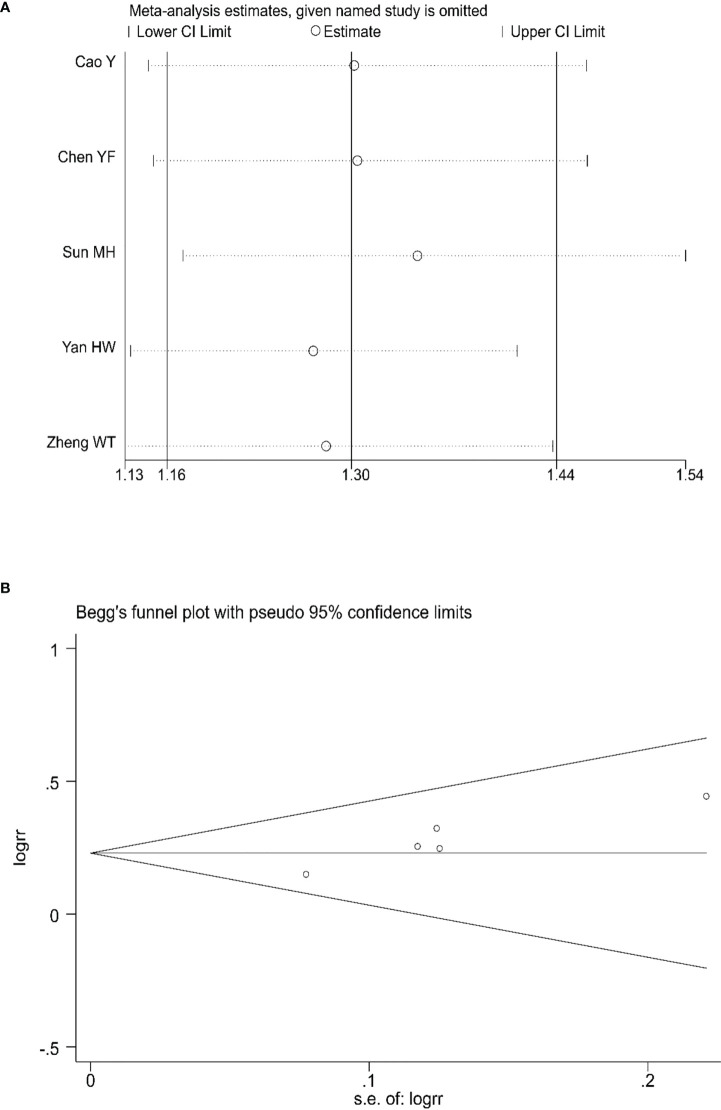
Sensitivity analysis **(A)** and Begg’s funnel plot **(B)** for the analysis of ORR. (Begg’s test: P= 0.221). ORR, objective response rate.

Sensitivity analyses were conducted to assess the impact of each study on the overall results by eliminating one study to assess whether there was heterogeneity. The results showed no evidence of heterogeneity in the CRR, PRR, or ORR (**Figures 7A**, **8A**, **9A**). Simultaneously, we evaluated the publication bias of the six included studies. A Begg’s funnel plot showed no significant publication bias in the CRR, PRR, or ORR (**Figures 7B**, **8B**, **9B**).

## 4 Discussion

EGFR belongs to a family of tyrosine kinase receptors and is expressed in the epithelial cell membrane. It triggers endogenous activation by binding to endogenous ligands such as epidermal growth factor (EGF), thereby maintaining the signal cascades of normal cells. Although EGFR is involved in the growth and development of cells and tissues under normal circumstances, its overexpression stimulates the growth and progression of tumour cells and promotes angiogenesis, invasion, and metastasis through major cascades such as Ras/Raf/MAPK, PIK-3/AKT, PLC-PKC, and STAT, which are related to the poor prognosis of malignant tumours (26, 27). Presently, two treatment methods, namely small-molecule tyrosine kinase inhibitors and monoclonal antibodies, are mainly used to inhibit abnormal EGFR-related signalling cascades. Monoclonal antibodies mainly bind to the extracellular region of receptors and inhibit dimerisation and autophosphorylation (28, 29). Several anti-EGFR monoclonal antibodies have been approved for cancer treatment, including cetuximab, panitumumab, nimotuzumab, and necitumumab. Nimotuzumab competes with EGFR to block downstream signalling pathways, inhibit tumour cell proliferation, promote tumour cell apoptosis, resist angiogenesis, and enhance the efficacy of radiotherapy or chemotherapy (10, 30, 31).

The clinical efficacy of nimotuzumab has been proven in a growing number of cancer types. A randomised phase III trial for locally advanced head and neck cancer showed that the nimotuzumab plus CRT had a significantly improved PFS, disease-free survival (DFS), and duration of locoregional control (LRC) compared with CRT alone (32). A randomised, open-label, phase III clinical trial evaluated the efficacy of nimotuzumab in combination with CRT versus CRT alone in the treatment of glioblastoma in 142 patients. The PFS at 12 months was 25.6% in the experimental group and 20.3% in the control group (33). Another study comparing the efficacy of CCRT with or without nimotuzumab in locally advanced squamous cell lung cancer showed a lower risk of brain metastases with nimotuzumab in combination with CCRT than with CCRT alone, although the OS and PFS were similar between the two groups (34). Although nimotuzumab is currently used less frequently for the treatment of CC, several studies have shown its reliable therapeutic effects. A retrospective study by Chen et al. evaluated the efficacy and adverse effects of nimotuzumab in combination with CCRT in patients with inoperable LACC (stages IIB- IIIB). The results showed that nimotuzumab in combination with CCRT for LACC resulted in a prolonged PFS and higher rates of complete remission without an increased incidence of adverse events (35). A single-arm study evaluating the efficacy of preoperative CCRT in combination with nimotuzumab in patients with LACC found that preoperative CCRT plus nimotuzumab achieved good local tumour control with acceptable toxicity (17).

Our meta-analysis systematically assessed the efficacy and safety of nimotuzumab for the treatment of CC. Six RCTs involving 393 patients were included. The results showed a significant improvement in CRR, ORR, and three-year survival in the nimotuzumab combination group compared with the CRT alone group. Of the six studies included, the study conducted by Tian et al. also investigated the one-year local recurrence rate in patients with CC, and found that the one-year local recurrence rate was significantly lower in the nimotuzumab combined with CRT treatment group than in the CRT treatment group (7.41% vs. 22.22%, P = 0.034 < 0.05) (23). Another study by Zheng et al. reported a three-year local recurrence rate that was significantly lower in the combined treatment group than in the control group (3.33% vs. 33.33%, P < 0.05) (25). Tian et al. reported both six-month and one-year survival rates, with a six-month survival rate of 96.29% vs. 85.18% and a one-year survival rate of 92.59% vs. 81.49% for the experimental and control groups, respectively, both with statistically significant differences (23). The study by Cao et al. showed a significantly higher three-year PFS rate in the nimotuzumab group (73.9% [34/46]) than in the CRT group (50.0% [23/46]) (P = 0.042 < 0.05) (20). These results show that nimotuzumab in combination with CRT has some advantages for the treatment of CC. However, the sample sizes included in the literature were small, and the subjects were all Chinese patients, whereas the efficacy of treatment for patients with CC in Western countries remains unknown.

A distinctive feature of nimotuzumab is its low toxicity compared with other anti-EGFR monoclonal antibodies (36, 37). The incidence of rash side effects associated with nimotuzumab was low, with only one mention of rash in our study, and no difference in the incidence of previous rashes between the experimental and control groups (11, 20). Our meta-analysis showed that nimotuzumab plus CRT did not increase the incidence of leukocytopenia, gastrointestinal reactions, radiation cystitis, or radiation proctitis in patients with CC compared to CRT treatment alone, which further supports the safety of nimotuzumab and provides a better quality of life for patients (38). Based on the above analysis, nimotuzumab combined with CRT may be an effective and safe treatment for CC.

### 4.1 Limitations

There were some limitations to our study. First, there were inconsistencies in the patients’ CRT regimens, and further research is needed to determine the optimal radiotherapy modality, radiotherapy dose, and combination chemotherapy regimens. Second, the sample sizes of the included studies were small, and larger randomised controlled clinical trials are needed to further assess the efficacy of nimotuzumab in combination with CRT for CC. Finally, it is unclear whether nimotuzumab in combination with CRT improves the OS and PFS rates in patients since most of the included studies did not include such outcome indicators.

## 5 Conclusion

In summary, our meta-analysis showed that the addition of nimotuzumab to CRT significantly improved the outcomes of patients with CC and did not increase the associated toxicities, which provides strong evidence that nimotuzumab is an effective treatment for CC. However, more prospective randomised controlled clinical trials are needed to fully explore the effectiveness of this treatment in patients with CC.

## Data availability statement

The data provided in this study are included in the article and **Supplementary Material**. The corresponding authors can be contacted for further information.

## Author contributions

YY, YX, and YiG conceived and designed the study. YY, JC, and MF performed the literature screening and data extraction. YY, YaG, XS, and DY were involved in quality evaluation and statistical analysis. YX revised the manuscript. All authors contributed to the article and approved the submitted version.

## Funding

This study was supported by the National Natural Science Foundation of China (Grant No. 81972845) and the Postgraduate Research & Practice Innovation Program of Jiangsu Province (SJCX22_1273).

## Conflict of interest

The authors declare that the research was conducted in the absence of any commercial or financial relationships that could be construed as a potential conflict of interest.

## Publisher’s note

All claims expressed in this article are solely those of the authors and do not necessarily represent those of their affiliated organizations, or those of the publisher, the editors and the reviewers. Any product that may be evaluated in this article, or claim that may be made by its manufacturer, is not guaranteed or endorsed by the publisher.
