# Supplementary material for: Nimotuzumab combined with chemoradiotherapy for the treatment of cervical cancer: A meta-analysis of randomized controlled trials
Source: Front Oncol. 2022 Oct 3;12:994726. doi: 10.3389/fonc.2022.994726 (PMC9573994; doi:10.3389/fonc.2022.994726)
Supplement: Supplementary file 1 [file DataSheet_1.docx]

Supplementary Material

# Supplementary materials caption

**TABLE S1** | Summary of the meta-analysis.

**TABLE S2** | Adverse effects.

**FIGURE S1** | Funnel plot for the analysis of CRR(A), PRR(B) and ORR(C).

**FIGURE S2** | Egger’s plot of the analysis of publication bias of CRR(A), PRR(B) and ORR(C).

**FIGURE S3** | Begg’s and Egger’s tests for publication bias of CRR(A), PRR(B) and ORR(C).

**Appendix S1** | Literature search strategies.

| **Outcomes and toxicity** | **Included study** | **Number of Experiment** | **Number of Control** | **Heterogeneity** | | **Meta-analysis model** | **Result of meta-analysis** | |
| --- | --- | --- | --- | --- | --- | --- | --- | --- |
|  |  |  |  | **P** | **I^2^** |  | **RR (95%CI)** | **P** |
| CRR | 4 | 140 | 139 | 0.98 | 0% | fixed-effects | 1.34(1.08-1.65) | 0.007 |
| PRR | 4 | 140 | 139 | 0.57 | 0% | fixed-effects | 1.15(0.76-1.75) | 0.51 |
| ORR | 5 | 170 | 169 | 0.56 | 0% | fixed-effects | 1.30(1.16-1.44) | <0.00001 |
| 3-year survival rate | 2 | 76 | 76 | 0.83 | 0% | fixed-effects | 1.27(1.06-1.51) | 0.008 |
| *Toxicity* |  |  |  |  |  |  |  |  |
| Leukocytopenia | 5 | 160 | 159 | 0.99 | 0% | fixed-effects | 0.89(0.66-1.20) | 0.45 |
| Gastrointestinal reaction | 6 | 197 | 196 | 0.93 | 0% | fixed-effects | 1.09(0.87-1.36) | 0.47 |
| Radiocystitis | 5 | 160 | 159 | 0.84 | 0% | fixed-effects | 0.92(0.43-1.95) | 0.82 |
| Radioproctitis | 4 | 139 | 139 | 0.93 | 0% | fixed-effects | 0.74(0.39-1.41) | 0.35 |

**TABLE S1** |Summary of the meta-analysis

| **Study** | **leukocytopenia** | |  | **gastrointestinal reaction** | |  | **radiocystitis** | |  | **radioproctitis** | |
| --- | --- | --- | --- | --- | --- | --- | --- | --- | --- | --- | --- |
|  | **Nimotuzumab + CRT group** | **CRT group** |  | **Nimotuzumab + CRT group** | **CRT group** |  | **Nimotuzumab + CRT group** | **CRT group** |  | **Nimotuzumab + CRT group** | **CRT group** |
| Cao Y  2019 (20) | 22 | 25 |  | 36 | 31 |  | 5 | 3 |  | 5 | 8 |
| Chen YF  2015 (21) | 12 | 13 |  | 15 | 14 |  | 3 | 3 |  | 4 | 5 |
| Sun MH  2020 (22) | NA | NA |  | 3 | 2 |  | NA | NA |  | NA | NA |
| Tian LC  2021 (23) | 4 | 5 |  | 2 | 1 |  | 1 | 2 |  | 1 | 2 |
| Yan HW  2021 (24) | 2 | 3 |  | 4 | 5 |  | 1 | 2 |  | NA | NA |
| Zheng WT  2018 (25) | 10 | 10 |  | 13 | 14 |  | 2 | 3 |  | 4 | 4 |

**TABLE S2** | Adverse effects.

NA: not available.

**A**


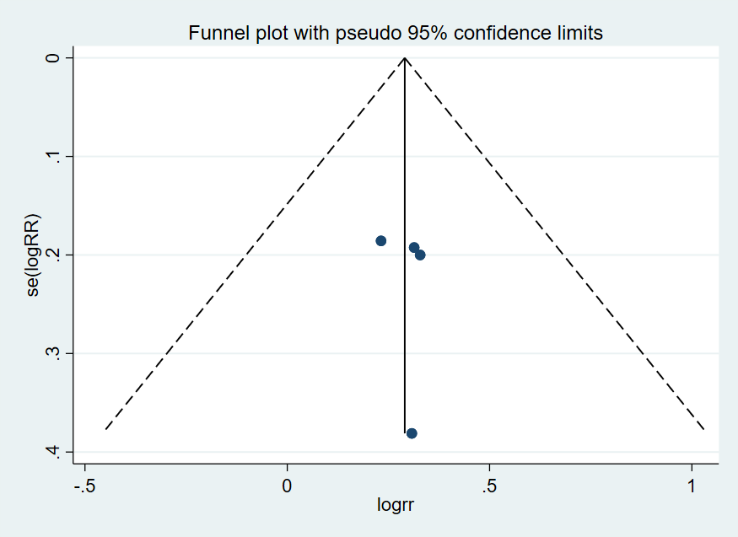


**B**


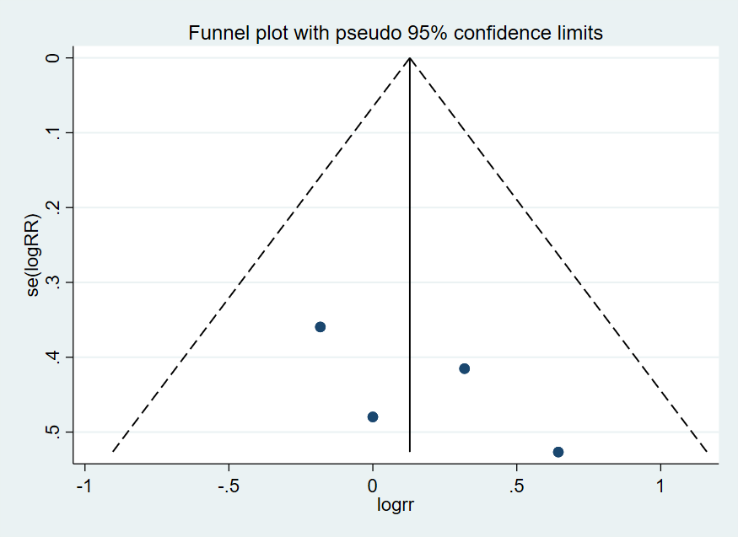


**C**


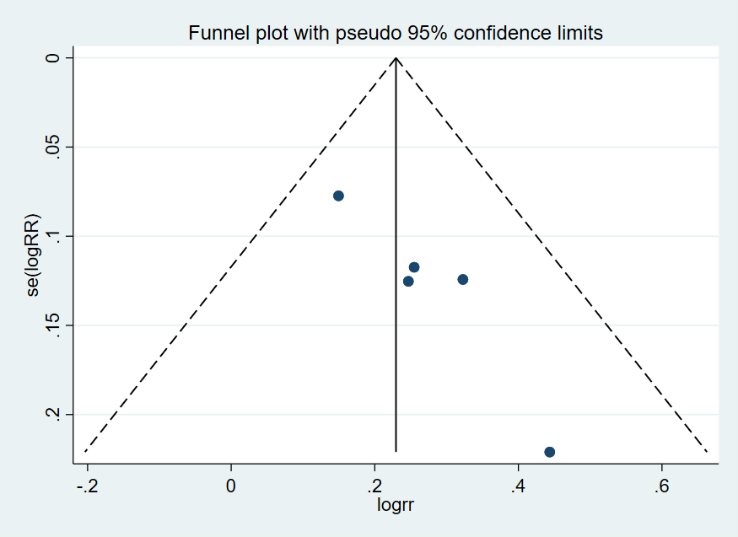


**FIGURE S1 |** Funnel plot for the analysis of CRR(A), PRR(B) and ORR(C).

CRR: complete remission rate; PRR: partial remission rate; ORR: objective response rate.

**A**

**B**

**C**

**FIGURE S2** | Egger’s plot for the analysis of CRR(A), PRR(B) and ORR(C).

CRR: complete remission rate; PRR: partial remission rate; ORR: objective response rate.

**A**


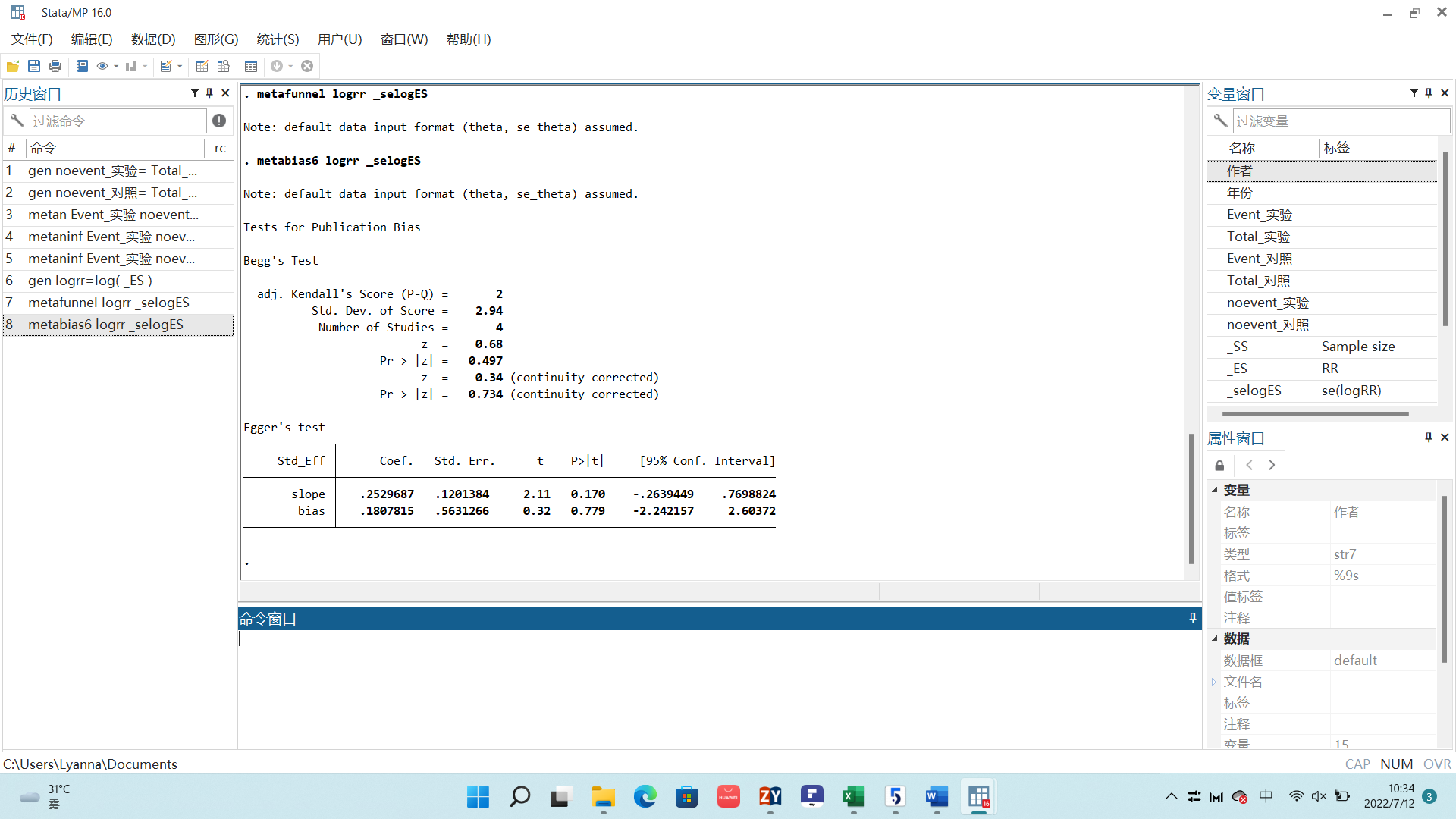


**B**


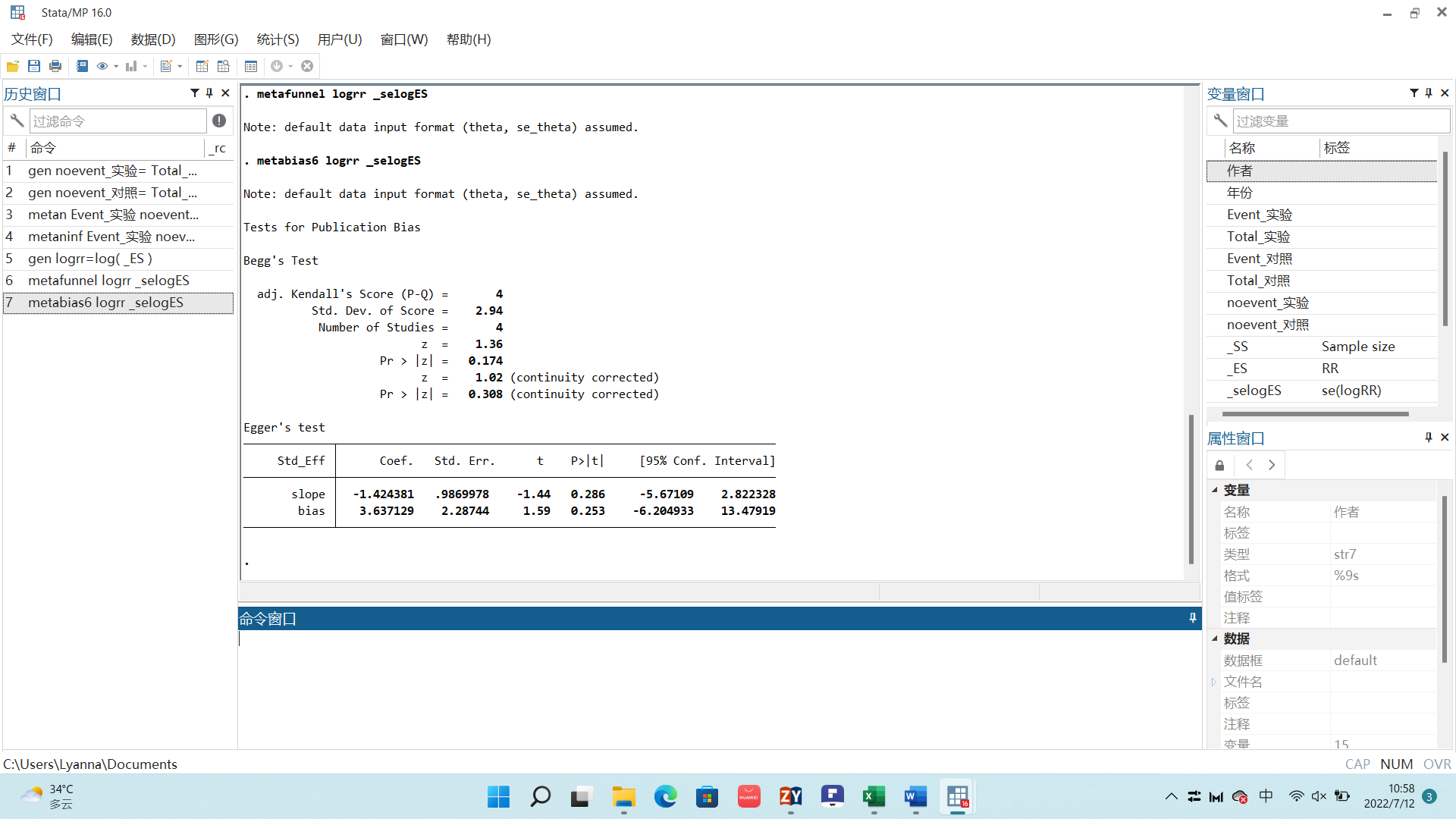


**C**


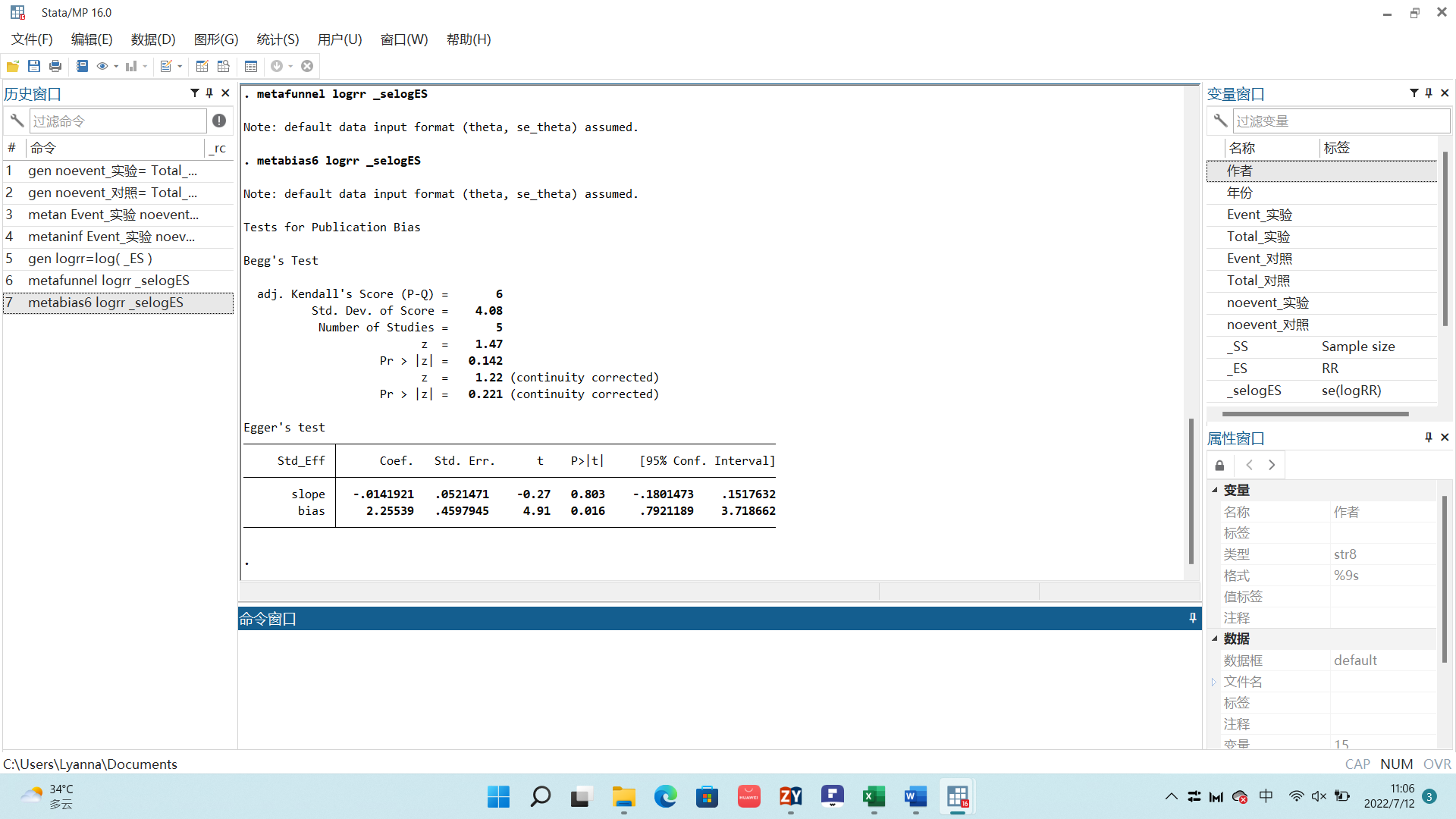


**FIGURE S3** | Begg’s and Egger’s tests for publication bias of CRR(A), PRR(B) and ORR(C).

CRR: complete remission rate; PRR: partial remission rate; ORR: objective response rate.

**PubMed**

| #15 | **((("Uterine Cervical Neoplasms"[Mesh]) OR ((((((((((((((((((((((((((Cervical Neoplasm, Uterine[Title/Abstract]) OR (Cervical Neoplasms, Uterine[Title/Abstract])) OR (Neoplasm, Uterine Cervical[Title/Abstract])) OR (Neoplasms, Uterine Cervical[Title/Abstract])) OR (Uterine Cervical Neoplasm[Title/Abstract])) OR (Neoplasms, Cervical[Title/Abstract])) OR (Cervical Neoplasms[Title/Abstract])) OR (Cervical Neoplasm[Title/Abstract])) OR (Neoplasm, Cervical[Title/Abstract])) OR (Neoplasms, Cervix[Title/Abstract])) OR (Cervix Neoplasms[Title/Abstract])) OR (Cervix Neoplasm[Title/Abstract])) OR (Neoplasm, Cervix[Title/Abstract])) OR (Cancer of the Uterine Cervix[Title/Abstract])) OR (Cancer of the Cervix[Title/Abstract])) OR (Cervical Cancer[Title/Abstract])) OR (Uterine Cervical Cancer[Title/Abstract])) OR (Cancer, Uterine Cervical[Title/Abstract])) OR (Cancers, Uterine Cervical[Title/Abstract])) OR (Cervical Cancer, Uterine[Title/Abstract])) OR (Cervical Cancers, Uterine[Title/Abstract])) OR (Uterine Cervical Cancers[Title/Abstract])) OR (Cancer of Cervix[Title/Abstract])) OR (Cervix Cancer[Title/Abstract])) OR (Cancer, Cervix[Title/Abstract])) OR (Cancers, Cervix[Title/Abstract]))) AND (((Nimotuzumab[Title/Abstract]) OR (theracim-HR3[Title/Abstract])) OR (Theraloc[Title/Abstract]))) AND (((("Chemoradiotherapy"[Mesh]) OR (((((((((((((((((((((((Chemoradiotherapies[Title/Abstract]) OR (Radiochemotherapy[Title/Abstract])) OR (Radiochemotherapies[Title/Abstract])) OR (Concurrent Chemoradiotherapy[Title/Abstract])) OR (Chemoradiotherapies, Concurrent[Title/Abstract])) OR (Chemoradiotherapy, Concurrent[Title/Abstract])) OR (Concurrent Chemoradiotherapies[Title/Abstract])) OR (Synchronous Chemoradiotherapy[Title/Abstract])) OR (Chemoradiotherapies, Synchronous[Title/Abstract])) OR (Chemoradiotherapy, Synchronous[Title/Abstract])) OR (Synchronous Chemoradiotherapies[Title/Abstract])) OR (Concurrent Radiochemotherapy[Title/Abstract])) OR (Concurrent Radiochemotherapies[Title/Abstract])) OR (Radiochemotherapies, Concurrent[Title/Abstract])) OR (Radiochemotherapy, Concurrent[Title/Abstract])) OR (Concomitant Chemoradiotherapy[Title/Abstract])) OR (Chemoradiotherapies, Concomitant[Title/Abstract])) OR (Chemoradiotherapy, Concomitant[Title/Abstract])) OR (Concomitant Chemoradiotherapies[Title/Abstract])) OR (Concomitant Radiochemotherapy[Title/Abstract])) OR (Concomitant Radiochemotherapies[Title/Abstract])) OR (Radiochemotherapies, Concomitant[Title/Abstract])) OR (Radiochemotherapy, Concomitant[Title/Abstract]))) OR (("Drug Therapy"[Mesh]) OR (((((((Therapy, Drug[Title/Abstract]) OR (Drug Therapies[Title/Abstract])) OR (Therapies, Drug[Title/Abstract])) OR (Chemotherapy[Title/Abstract])) OR (Chemotherapies[Title/Abstract])) OR (Pharmacotherapy[Title/Abstract])) OR (Pharmacotherapies[Title/Abstract])))) OR (("Radiotherapy"[Mesh]) OR ((((((((((((((((((Radiotherapies[Title/Abstract]) OR (Radiation Therapy[Title/Abstract])) OR (Radiation Therapies[Title/Abstract])) OR (Therapies, Radiation[Title/Abstract])) OR (Therapy, Radiation[Title/Abstract])) OR (Radiation Treatment[Title/Abstract])) OR (Radiation Treatments[Title/Abstract])) OR (Treatment, Radiation[Title/Abstract])) OR (Radiotherapy, Targeted[Title/Abstract])) OR (Radiotherapies, Targeted[Title/Abstract])) OR (Targeted Radiotherapies[Title/Abstract])) OR (Targeted Radiotherapy[Title/Abstract])) OR (Targeted Radiation Therapy[Title/Abstract])) OR (Radiation Therapies, Targeted[Title/Abstract])) OR (Targeted Radiation Therapies[Title/Abstract])) OR (Therapies, Targeted Radiation[Title/Abstract])) OR (Therapy, Targeted Radiation[Title/Abstract])) OR (Radiation Therapy, Targeted[Title/Abstract]))))** | 7 |
| --- | --- | --- |
| #14 | **((("Chemoradiotherapy"[Mesh]) OR (((((((((((((((((((((((Chemoradiotherapies[Title/Abstract]) OR (Radiochemotherapy[Title/Abstract])) OR (Radiochemotherapies[Title/Abstract])) OR (Concurrent Chemoradiotherapy[Title/Abstract])) OR (Chemoradiotherapies, Concurrent[Title/Abstract])) OR (Chemoradiotherapy, Concurrent[Title/Abstract])) OR (Concurrent Chemoradiotherapies[Title/Abstract])) OR (Synchronous Chemoradiotherapy[Title/Abstract])) OR (Chemoradiotherapies, Synchronous[Title/Abstract])) OR (Chemoradiotherapy, Synchronous[Title/Abstract])) OR (Synchronous Chemoradiotherapies[Title/Abstract])) OR (Concurrent Radiochemotherapy[Title/Abstract])) OR (Concurrent Radiochemotherapies[Title/Abstract])) OR (Radiochemotherapies, Concurrent[Title/Abstract])) OR (Radiochemotherapy, Concurrent[Title/Abstract])) OR (Concomitant Chemoradiotherapy[Title/Abstract])) OR (Chemoradiotherapies, Concomitant[Title/Abstract])) OR (Chemoradiotherapy, Concomitant[Title/Abstract])) OR (Concomitant Chemoradiotherapies[Title/Abstract])) OR (Concomitant Radiochemotherapy[Title/Abstract])) OR (Concomitant Radiochemotherapies[Title/Abstract])) OR (Radiochemotherapies, Concomitant[Title/Abstract])) OR (Radiochemotherapy, Concomitant[Title/Abstract]))) OR (("Drug Therapy"[Mesh]) OR (((((((Therapy, Drug[Title/Abstract]) OR (Drug Therapies[Title/Abstract])) OR (Therapies, Drug[Title/Abstract])) OR (Chemotherapy[Title/Abstract])) OR (Chemotherapies[Title/Abstract])) OR (Pharmacotherapy[Title/Abstract])) OR (Pharmacotherapies[Title/Abstract])))) OR (("Radiotherapy"[Mesh]) OR ((((((((((((((((((Radiotherapies[Title/Abstract]) OR (Radiation Therapy[Title/Abstract])) OR (Radiation Therapies[Title/Abstract])) OR (Therapies, Radiation[Title/Abstract])) OR (Therapy, Radiation[Title/Abstract])) OR (Radiation Treatment[Title/Abstract])) OR (Radiation Treatments[Title/Abstract])) OR (Treatment, Radiation[Title/Abstract])) OR (Radiotherapy, Targeted[Title/Abstract])) OR (Radiotherapies, Targeted[Title/Abstract])) OR (Targeted Radiotherapies[Title/Abstract])) OR (Targeted Radiotherapy[Title/Abstract])) OR (Targeted Radiation Therapy[Title/Abstract])) OR (Radiation Therapies, Targeted[Title/Abstract])) OR (Targeted Radiation Therapies[Title/Abstract])) OR (Therapies, Targeted Radiation[Title/Abstract])) OR (Therapy, Targeted Radiation[Title/Abstract])) OR (Radiation Therapy, Targeted[Title/Abstract])))** | [1,945,822](https://pubmed.ncbi.nlm.nih.gov/?term=longquery733aed42d4273c41416f&sort=&size=50) |
| #13 | **("Radiotherapy"[Mesh]) OR ((((((((((((((((((Radiotherapies[Title/Abstract]) OR (Radiation Therapy[Title/Abstract])) OR (Radiation Therapies[Title/Abstract])) OR (Therapies, Radiation[Title/Abstract])) OR (Therapy, Radiation[Title/Abstract])) OR (Radiation Treatment[Title/Abstract])) OR (Radiation Treatments[Title/Abstract])) OR (Treatment, Radiation[Title/Abstract])) OR (Radiotherapy, Targeted[Title/Abstract])) OR (Radiotherapies, Targeted[Title/Abstract])) OR (Targeted Radiotherapies[Title/Abstract])) OR (Targeted Radiotherapy[Title/Abstract])) OR (Targeted Radiation Therapy[Title/Abstract])) OR (Radiation Therapies, Targeted[Title/Abstract])) OR (Targeted Radiation Therapies[Title/Abstract])) OR (Therapies, Targeted Radiation[Title/Abstract])) OR (Therapy, Targeted Radiation[Title/Abstract])) OR (Radiation Therapy, Targeted[Title/Abstract]))** | 255684 |
| #12 | **(((((((((((((((((Radiotherapies[Title/Abstract]) OR (Radiation Therapy[Title/Abstract])) OR (Radiation Therapies[Title/Abstract])) OR (Therapies, Radiation[Title/Abstract])) OR (Therapy, Radiation[Title/Abstract])) OR (Radiation Treatment[Title/Abstract])) OR (Radiation Treatments[Title/Abstract])) OR (Treatment, Radiation[Title/Abstract])) OR (Radiotherapy, Targeted[Title/Abstract])) OR (Radiotherapies, Targeted[Title/Abstract])) OR (Targeted Radiotherapies[Title/Abstract])) OR (Targeted Radiotherapy[Title/Abstract])) OR (Targeted Radiation Therapy[Title/Abstract])) OR (Radiation Therapies, Targeted[Title/Abstract])) OR (Targeted Radiation Therapies[Title/Abstract])) OR (Therapies, Targeted Radiation[Title/Abstract])) OR (Therapy, Targeted Radiation[Title/Abstract])) OR (Radiation Therapy, Targeted[Title/Abstract])** | 95503 |
| #11 | **"Radiotherapy"[Mesh]** Sort by: **Most Recent** | 200471 |
| #10 | **("Drug Therapy"[Mesh]) OR (((((((Therapy, Drug[Title/Abstract]) OR (Drug Therapies[Title/Abstract])) OR (Therapies, Drug[Title/Abstract])) OR (Chemotherapy[Title/Abstract])) OR (Chemotherapies[Title/Abstract])) OR (Pharmacotherapy[Title/Abstract])) OR (Pharmacotherapies[Title/Abstract]))** | 1760353 |
| #9 | **((((((Therapy, Drug[Title/Abstract]) OR (Drug Therapies[Title/Abstract])) OR (Therapies, Drug[Title/Abstract])) OR (Chemotherapy[Title/Abstract])) OR (Chemotherapies[Title/Abstract])) OR (Pharmacotherapy[Title/Abstract])) OR (Pharmacotherapies[Title/Abstract])** | 456184 |
| #8 | **"Drug Therapy"[Mesh]** Sort by: **Most Recent** | 1457978 |
| #7 | **("Chemoradiotherapy"[Mesh]) OR (((((((((((((((((((((((Chemoradiotherapies[Title/Abstract]) OR (Radiochemotherapy[Title/Abstract])) OR (Radiochemotherapies[Title/Abstract])) OR (Concurrent Chemoradiotherapy[Title/Abstract])) OR (Chemoradiotherapies, Concurrent[Title/Abstract])) OR (Chemoradiotherapy, Concurrent[Title/Abstract])) OR (Concurrent Chemoradiotherapies[Title/Abstract])) OR (Synchronous Chemoradiotherapy[Title/Abstract])) OR (Chemoradiotherapies, Synchronous[Title/Abstract])) OR (Chemoradiotherapy, Synchronous[Title/Abstract])) OR (Synchronous Chemoradiotherapies[Title/Abstract])) OR (Concurrent Radiochemotherapy[Title/Abstract])) OR (Concurrent Radiochemotherapies[Title/Abstract])) OR (Radiochemotherapies, Concurrent[Title/Abstract])) OR (Radiochemotherapy, Concurrent[Title/Abstract])) OR (Concomitant Chemoradiotherapy[Title/Abstract])) OR (Chemoradiotherapies, Concomitant[Title/Abstract])) OR (Chemoradiotherapy, Concomitant[Title/Abstract])) OR (Concomitant Chemoradiotherapies[Title/Abstract])) OR (Concomitant Radiochemotherapy[Title/Abstract])) OR (Concomitant Radiochemotherapies[Title/Abstract])) OR (Radiochemotherapies, Concomitant[Title/Abstract])) OR (Radiochemotherapy, Concomitant[Title/Abstract]))** | 27353 |
| #6 | **((((((((((((((((((((((Chemoradiotherapies[Title/Abstract]) OR (Radiochemotherapy[Title/Abstract])) OR (Radiochemotherapies[Title/Abstract])) OR (Concurrent Chemoradiotherapy[Title/Abstract])) OR (Chemoradiotherapies, Concurrent[Title/Abstract])) OR (Chemoradiotherapy, Concurrent[Title/Abstract])) OR (Concurrent Chemoradiotherapies[Title/Abstract])) OR (Synchronous Chemoradiotherapy[Title/Abstract])) OR (Chemoradiotherapies, Synchronous[Title/Abstract])) OR (Chemoradiotherapy, Synchronous[Title/Abstract])) OR (Synchronous Chemoradiotherapies[Title/Abstract])) OR (Concurrent Radiochemotherapy[Title/Abstract])) OR (Concurrent Radiochemotherapies[Title/Abstract])) OR (Radiochemotherapies, Concurrent[Title/Abstract])) OR (Radiochemotherapy, Concurrent[Title/Abstract])) OR (Concomitant Chemoradiotherapy[Title/Abstract])) OR (Chemoradiotherapies, Concomitant[Title/Abstract])) OR (Chemoradiotherapy, Concomitant[Title/Abstract])) OR (Concomitant Chemoradiotherapies[Title/Abstract])) OR (Concomitant Radiochemotherapy[Title/Abstract])) OR (Concomitant Radiochemotherapies[Title/Abstract])) OR (Radiochemotherapies, Concomitant[Title/Abstract])) OR (Radiochemotherapy, Concomitant[Title/Abstract])** | [15017](https://www.embase.com/) |
| #5 | **"Chemoradiotherapy"[Mesh]** Sort by: **Most Recent** | 18356 |
| #4 | **((Nimotuzumab[Title/Abstract]) OR (theracim-HR3[Title/Abstract])) OR (Theraloc[Title/Abstract])** | 336 |
| #3 | **("Uterine Cervical Neoplasms"[Mesh]) OR ((((((((((((((((((((((((((Cervical Neoplasm, Uterine[Title/Abstract]) OR (Cervical Neoplasms, Uterine[Title/Abstract])) OR (Neoplasm, Uterine Cervical[Title/Abstract])) OR (Neoplasms, Uterine Cervical[Title/Abstract])) OR (Uterine Cervical Neoplasm[Title/Abstract])) OR (Neoplasms, Cervical[Title/Abstract])) OR (Cervical Neoplasms[Title/Abstract])) OR (Cervical Neoplasm[Title/Abstract])) OR (Neoplasm, Cervical[Title/Abstract])) OR (Neoplasms, Cervix[Title/Abstract])) OR (Cervix Neoplasms[Title/Abstract])) OR (Cervix Neoplasm[Title/Abstract])) OR (Neoplasm, Cervix[Title/Abstract])) OR (Cancer of the Uterine Cervix[Title/Abstract])) OR (Cancer of the Cervix[Title/Abstract])) OR (Cervical Cancer[Title/Abstract])) OR (Uterine Cervical Cancer[Title/Abstract])) OR (Cancer, Uterine Cervical[Title/Abstract])) OR (Cancers, Uterine Cervical[Title/Abstract])) OR (Cervical Cancer, Uterine[Title/Abstract])) OR (Cervical Cancers, Uterine[Title/Abstract])) OR (Uterine Cervical Cancers[Title/Abstract])) OR (Cancer of Cervix[Title/Abstract])) OR (Cervix Cancer[Title/Abstract])) OR (Cancer, Cervix[Title/Abstract])) OR (Cancers, Cervix[Title/Abstract]))** | 105721 |
| #2 | **(((((((((((((((((((((((((Cervical Neoplasm, Uterine[Title/Abstract]) OR (Cervical Neoplasms, Uterine[Title/Abstract])) OR (Neoplasm, Uterine Cervical[Title/Abstract])) OR (Neoplasms, Uterine Cervical[Title/Abstract])) OR (Uterine Cervical Neoplasm[Title/Abstract])) OR (Neoplasms, Cervical[Title/Abstract])) OR (Cervical Neoplasms[Title/Abstract])) OR (Cervical Neoplasm[Title/Abstract])) OR (Neoplasm, Cervical[Title/Abstract])) OR (Neoplasms, Cervix[Title/Abstract])) OR (Cervix Neoplasms[Title/Abstract])) OR (Cervix Neoplasm[Title/Abstract])) OR (Neoplasm, Cervix[Title/Abstract])) OR (Cancer of the Uterine Cervix[Title/Abstract])) OR (Cancer of the Cervix[Title/Abstract])) OR (Cervical Cancer[Title/Abstract])) OR (Uterine Cervical Cancer[Title/Abstract])) OR (Cancer, Uterine Cervical[Title/Abstract])) OR (Cancers, Uterine Cervical[Title/Abstract])) OR (Cervical Cancer, Uterine[Title/Abstract])) OR (Cervical Cancers, Uterine[Title/Abstract])) OR (Uterine Cervical Cancers[Title/Abstract])) OR (Cancer of Cervix[Title/Abstract])) OR (Cervix Cancer[Title/Abstract])) OR (Cancer, Cervix[Title/Abstract])) OR (Cancers, Cervix[Title/Abstract])** | 81541 |
| #1 | **"Uterine Cervical Neoplasms"[Mesh]** Sort by: **Most Recent** | 80968 |

**EMBASE**

| #7 | #1 AND #2 AND #6 | 20 |
| --- | --- | --- |
| #6 | #3 OR #4 OR #5 | [1,056,718](https://www.embase.com/) |
| #5 | 'radiotherapy':ab,ti OR 'radiotherapies':ab,ti OR 'radiation therapy':ab,ti OR 'radiation therapies':ab,ti OR 'therapies, radiation':ab,ti OR 'therapy, radiation':ab,ti OR 'radiation treatment':ab,ti OR 'radiation treatments':ab,ti OR 'treatment, radiation':ab,ti OR 'radiotherapy, targeted':ab,ti OR 'radiotherapies, targeted':ab,ti OR 'targeted radiotherapies':ab,ti OR 'targeted radiotherapy':ab,ti OR 'targeted radiation therapy':ab,ti OR 'radiation therapies, targeted':ab,ti OR 'targeted radiation therapies':ab,ti OR 'therapies, targeted radiation':ab,ti OR 'therapy, targeted radiation':ab,ti OR 'radiation therapy, targeted':ab,ti | 400891 |
| #4 | 'drug therapy':ab,ti OR 'therapy, drug':ab,ti OR 'drug therapies':ab,ti OR 'therapies, drug':ab,ti OR 'chemotherapy':ab,ti OR 'chemotherapies':ab,ti OR 'pharmacotherapy':ab,ti OR 'pharmacotherapies':ab,ti | 768790 |
| #3 | 'chemoradiotherapy':ab,ti OR 'chemoradiotherapies':ab,ti OR 'radiochemotherapy':ab,ti OR 'radiochemotherapies':ab,ti OR 'concurrent chemoradiotherapy':ab,ti OR 'chemoradiotherapies, concurrent':ab,ti OR 'chemoradiotherapy, concurrent':ab,ti OR 'concurrent chemoradiotherapies':ab,ti OR 'synchronous chemoradiotherapy':ab,ti OR 'chemoradiotherapies, synchronous':ab,ti OR 'chemoradiotherapy, synchronous':ab,ti OR 'synchronous chemoradiotherapies':ab,ti OR 'concurrent radiochemotherapy':ab,ti OR 'concurrent radiochemotherapies':ab,ti OR 'radiochemotherapies, concurrent':ab,ti OR 'radiochemotherapy, concurrent':ab,ti OR 'concomitant chemoradiotherapy':ab,ti OR 'chemoradiotherapies, concomitant':ab,ti OR 'chemoradiotherapy, concomitant':ab,ti OR 'concomitant chemoradiotherapies':ab,ti OR 'concomitant radiochemotherapy':ab,ti OR 'concomitant radiochemotherapies':ab,ti OR 'radiochemotherapies, concomitant':ab,ti OR 'radiochemotherapy, concomitant':ab,ti | 45683 |
| #2 | 'nimotuzumab':ab,ti OR 'theracim-hr3':ab,ti OR 'theraloc':ab,ti | 673 |
| #1 | 'uterine cervical neoplasms':ab,ti OR 'cervical neoplasm, uterine':ab,ti OR 'cervical neoplasms, uterine':ab,ti OR 'neoplasm, uterine cervical':ab,ti OR 'neoplasms, uterine cervical':ab,ti OR 'uterine cervical neoplasm':ab,ti OR 'neoplasms, cervical':ab,ti OR 'cervical neoplasms':ab,ti OR 'cervical neoplasm':ab,ti OR 'neoplasm, cervical':ab,ti OR 'neoplasms, cervix':ab,ti OR 'cervix neoplasms':ab,ti OR 'cervix neoplasm':ab,ti OR 'neoplasm, cervix':ab,ti OR 'cancer of the uterine cervix':ab,ti OR 'cancer of the cervix':ab,ti OR 'cervical cancer':ab,ti OR 'uterine cervical cancer':ab,ti OR 'cancer, uterine cervical':ab,ti OR 'cancers, uterine cervical':ab,ti OR 'cervical cancer, uterine':ab,ti OR 'cervical cancers, uterine':ab,ti OR 'uterine cervical cancers':ab,ti OR 'cancer of cervix':ab,ti OR 'cervix cancer':ab,ti OR 'cancer, cervix':ab,ti OR 'cancers, cervix':ab,ti | 79225 |

Web of Science

| #7 | **#1 AND #2 AND #6** | 21 |
| --- | --- | --- |
| #6 | #3 OR #4 OR #5 | [6,903,087](https://www.webofscience.com/wos/alldb/summary/15991196-527d-4c29-8f7e-dd3aa7af1fc9-2c0b4d8e/relevance/1) |
| #5 | **TS= (Radiotherapy OR Radiotherapies OR Radiation Therapy OR Radiation Therapies OR Therapies, Radiation OR Therapy, Radiation OR Radiation Treatment OR Radiation Treatments OR Treatment, Radiation OR Radiotherapy, Targeted OR Radiotherapies, Targeted OR Targeted Radiotherapies OR Targeted Radiotherapy OR Targeted Radiation Therapy OR Radiation Therapies, Targeted OR Targeted Radiation Therapies OR Therapies, Targeted Radiation OR Therapy, Targeted Radiation OR Radiation Therapy, Targeted)** | [1,300,610](https://www.webofscience.com/wos/alldb/summary/88016365-7cae-4e09-bfb7-d2acd11ac8e1-2c0b4796/relevance/1) |
| #4 | **TS= (Drug Therapy OR Therapy, Drug OR Drug Therapies OR Therapies, Drug OR Chemotherapy OR Chemotherapies OR Pharmacotherapy OR Pharmacotherapies)** | [6,017,395](https://www.webofscience.com/wos/alldb/summary/aa74737a-8462-4c77-b702-70a91e67045a-2c0b436d/relevance/1) |
| #3 | **TS= (Chemoradiotherapy OR Radiochemotherapy OR Radiochemotherapies OR Concurrent Chemoradiotherapy OR Chemoradiotherapies, Concurrent OR Chemoradiotherapy, Concurrent OR Concurrent Chemoradiotherapies OR Synchronous Chemoradiotherapy OR Chemoradiotherapies, Synchronous OR Chemoradiotherapy, Synchronous OR Synchronous Chemoradiotherapies OR Concurrent Radiochemotherapy OR Concurrent Radiochemotherapies OR Radiochemotherapies, Concurrent OR Radiochemotherapy, Concurrent OR Concomitant Chemoradiotherapy OR Chemoradiotherapies, Concomitant OR Chemoradiotherapy, Concomitant OR Concomitant Chemoradiotherapies OR Concomitant Radiochemotherapy OR Concomitant Radiochemotherapies OR Radiochemotherapies, Concomitant OR Radiochemotherapy, Concomitant)** | 53350 |
| #2 | **TS= (Nimotuzumab OR theracim-HR3 OR Theraloc)** | 677 |
| #1 | **TS= (Uterine Cervical Neoplasms OR Cervical Neoplasm, Uterine OR Cervical Neoplasms, Uterine OR Neoplasm, Uterine Cervical OR Neoplasms, Uterine Cervical OR Uterine Cervical Neoplasm OR Neoplasms, Cervical OR Cervical Neoplasms OR Cervical Neoplasm OR Neoplasm, Cervical OR Neoplasms, Cervix OR Cervix Neoplasms OR Cervix Neoplasm OR Neoplasm, Cervix OR Cancer of the Uterine Cervix OR Cancer of the Cervix OR Cervical Cancer OR Uterine Cervical Cancer OR Cancer, Uterine Cervical OR Cancers, Uterine Cervical OR Cervical Cancer, Uterine OR Cervical Cancers, Uterine OR Uterine Cervical Cancers OR Cancer of Cervix OR Cervix Cancer OR Cancer, Cervix OR Cancers, Cervix)** | 226463 |

Cochrane Library

| #1 | (Uterine Cervical Neoplasms):ti,ab,kw OR (Cervical Neoplasm, Uterine):ti,ab,kw OR (Cervical Neoplasms, Uterine):ti,ab,kw OR (Neoplasm, Uterine Cervical):ti,ab,kw OR (Neoplasms, Uterine Cervical):ti,ab,kw OR (Uterine Cervical Neoplasm):ti,ab,kw OR (Neoplasms, Cervical):ti,ab,kw OR (Cervical Neoplasms):ti,ab,kw OR (Cervical Neoplasm):ti,ab,kw OR (Neoplasm, Cervical):ti,ab,kw OR (Neoplasms, Cervix):ti,ab,kw OR (Cervix Neoplasms):ti,ab,kw OR (Cervix Neoplasm):ti,ab,kw OR (Neoplasm, Cervix):ti,ab,kw OR (Cancer of the Uterine Cervix):ti,ab,kw OR (Cancer of the Cervix):ti,ab,kw OR (Cervical Cancer):ti,ab,kw OR (Uterine Cervical Cancer):ti,ab,kw OR (Cancer, Uterine Cervical):ti,ab,kw OR (Cancers, Uterine Cervical):ti,ab,kw OR (Cervical Cancer, Uterine):ti,ab,kw OR (Cervical Cancers, Uterine):ti,ab,kw OR (Uterine Cervical Cancers):ti,ab,kw OR (Cancer of Cervix):ti,ab,kw OR (Cervix Cancer):ti,ab,kw OR (Cancer, Cervix):ti,ab,kw OR (Cancers, Cervix):ti,ab,kw | 6803 |
| --- | --- | --- |
| #2 | (Nimotuzumab):ti,ab,kw OR (theracim-HR3):ti,ab,kw OR (Theraloc):ti,ab,kw | [165](https://www.webofscience.com/wos/alldb/summary/15991196-527d-4c29-8f7e-dd3aa7af1fc9-2c0b4d8e/relevance/1) |
| #3 | (Chemoradiotherapy):ti,ab,kw OR (Chemoradiotherapies):ti,ab,kw OR (Radiochemotherapy):ti,ab,kw OR (Radiochemotherapies):ti,ab,kw OR (Concurrent Chemoradiotherapy):ti,ab,kw OR (Chemoradiotherapies, Concurrent):ti,ab,kw OR (Chemoradiotherapy, Concurrent):ti,ab,kw OR (Concurrent Chemoradiotherapies):ti,ab,kw OR (Synchronous Chemoradiotherapy):ti,ab,kw OR (Chemoradiotherapies, Synchronous):ti,ab,kw OR (Chemoradiotherapy, Synchronous):ti,ab,kw OR (Synchronous Chemoradiotherapies):ti,ab,kw OR (Concurrent Radiochemotherapy):ti,ab,kw OR (Concurrent Radiochemotherapies):ti,ab,kw OR (Radiochemotherapies, Concurrent):ti,ab,kw OR (Radiochemotherapy, Concurrent):ti,ab,kw OR (Concomitant Chemoradiotherapy):ti,ab,kw OR (Chemoradiotherapies, Concomitant):ti,ab,kw OR (Chemoradiotherapy, Concomitant):ti,ab,kw OR (Concomitant Chemoradiotherapies):ti,ab,kw OR (Concomitant Radiochemotherapy):ti,ab,kw OR (Concomitant Radiochemotherapies):ti,ab,kw OR (Radiochemotherapies, Concomitant):ti,ab,kw OR (Radiochemotherapy, Concomitant):ti,ab,kw | [7897](https://www.webofscience.com/wos/alldb/summary/88016365-7cae-4e09-bfb7-d2acd11ac8e1-2c0b4796/relevance/1) |
| #4 | (Drug Therapy):ti,ab,kw OR (Therapy, Drug):ti,ab,kw OR (Drug Therapies):ti,ab,kw OR (Therapies, Drug):ti,ab,kw OR (Chemotherapy):ti,ab,kw OR (Chemotherapies):ti,ab,kw OR (Pharmacotherapy):ti,ab,kw OR (Pharmacotherapies):ti,ab,kw | [515149](https://www.webofscience.com/wos/alldb/summary/aa74737a-8462-4c77-b702-70a91e67045a-2c0b436d/relevance/1) |
| #5 | (Radiotherapy):ti,ab,kw OR (Radiotherapies):ti,ab,kw OR (Radiation Therapy):ti,ab,kw OR (Radiation Therapies):ti,ab,kw OR (Therapies, Radiation):ti,ab,kw OR (Therapy, Radiation):ti,ab,kw OR (Radiation Treatment):ti,ab,kw OR (Radiation Treatments):ti,ab,kw OR (Treatment, Radiation):ti,ab,kw OR (Radiotherapy, Targeted):ti,ab,kw OR (Radiotherapies, Targeted):ti,ab,kw OR (Targeted Radiotherapies):ti,ab,kw OR (Targeted Radiotherapy):ti,ab,kw OR (Targeted Radiation Therapy):ti,ab,kw OR (Radiation Therapies, Targeted):ti,ab,kw OR (Targeted Radiation Therapies):ti,ab,kw OR (Therapies, Targeted Radiation):ti,ab,kw OR (Therapy, Targeted Radiation):ti,ab,kw OR (Radiation Therapy, Targeted):ti,ab,kw | 46128 |
| #6 | #3 OR #4 OR #5 | 536290 |
| #7 | **#1 AND #2 AND #6** | 11 |

**Appendix S1** | Literature search strategies.

**
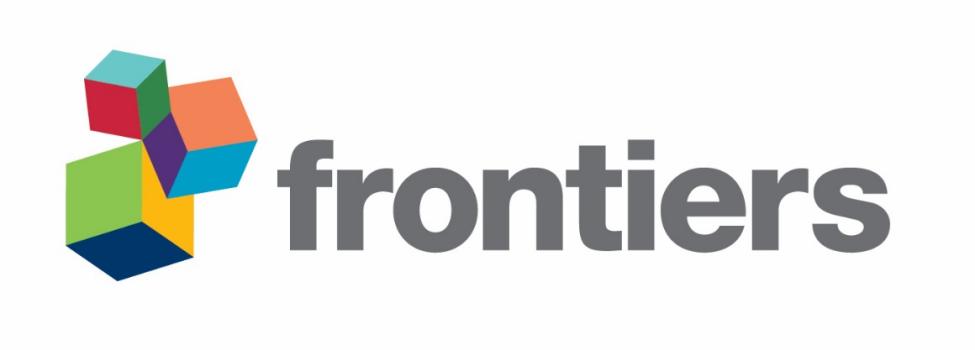
**
